# Immunological hyporesponsiveness in tuberculosis: The role of mycobacterial glycolipids

**DOI:** 10.3389/fimmu.2022.1035122

**Published:** 2022-12-02

**Authors:** Margarida Correia-Neves, Jérôme Nigou, Zaynab Mousavian, Christopher Sundling, Gunilla Källenius

**Affiliations:** ^1^ Life and Health Sciences Research Institute, School of Medicine, University of Minho, Braga, Portugal; ^2^ Life and Health Sciences Research Institute/Biomaterials, Biodegradables and Biomimetics Research Group (ICVS/3B's), Portuguese (PT) Government Associate Laboratory, Braga, Portugal; ^3^ Division of Infectious Diseases, Department of Medicine Solna, Karolinska Institutet, Stockholm, Sweden; ^4^ Institut de Pharmacologie et de Biologie Structurale, Université de Toulouse, Centre National de la Recherche Scientifique (CNRS), Université Paul Sabatier, Toulouse, France; ^5^ School of Mathematics, Statistics, and Computer Science, College of Science, University of Tehran, Tehran, Iran; ^6^ Center for Molecular Medicine, Karolinska Institutet, Stockholm, Sweden; ^7^ Department of Infectious Diseases, Karolinska University Hospital, Stockholm, Sweden

**Keywords:** glycolipid, *Mycobacterium*, tuberculosis, immunological tolerance, latency, lipoarabinomannan

## Abstract

Glycolipids constitute a major part of the cell envelope of *Mycobacterium tuberculosis* (Mtb). They are potent immunomodulatory molecules recognized by several immune receptors like pattern recognition receptors such as TLR2, DC-SIGN and Dectin-2 on antigen-presenting cells and by T cell receptors on T lymphocytes. The Mtb glycolipids lipoarabinomannan (LAM) and its biosynthetic relatives, phosphatidylinositol mannosides (PIMs) and lipomannan (LM), as well as other Mtb glycolipids, such as phenolic glycolipids and sulfoglycolipids have the ability to modulate the immune response, stimulating or inhibiting a pro-inflammatory response. We explore here the downmodulating effect of Mtb glycolipids. A great proportion of the studies used *in vitro* approaches although *in vivo* infection with Mtb might also lead to a dampening of myeloid cell and T cell responses to Mtb glycolipids. This dampened response has been explored *ex vivo* with immune cells from peripheral blood from Mtb-infected individuals and in mouse models of infection. In addition to the dampening of the immune response caused by Mtb glycolipids, we discuss the hyporesponse to Mtb glycolipids caused by prolonged Mtb infection and/or exposure to Mtb antigens. Hyporesponse to LAM has been observed in myeloid cells from individuals with active and latent tuberculosis (TB). For some myeloid subsets, this effect is stronger in latent versus active TB. Since the immune response in individuals with latent TB represents a more protective profile compared to the one in patients with active TB, this suggests that downmodulation of myeloid cell functions by Mtb glycolipids may be beneficial for the host and protect against active TB disease. The mechanisms of this downmodulation, including tolerance through epigenetic modifications, are only partly explored.

## 1 Introduction

Tuberculosis (TB), caused by *Mycobacterium tuberculosis* (Mtb), is a chronic disease, causing approximately 1.6 million deaths per year. Morbidity is also significant with 10 million people estimated to be affected by the disease each year and the threat to global health is further amplified by the emergence of approximately 600,000 cases of drug-resistant TB each year, as well as by the effects of the Covid-19 pandemic, estimated to have caused an increase of about 100,000 in the global number of TB deaths between 2019 and 2020 (1, 2).

Exposure to Mtb may lead to active TB, latent TB, or clearance of Mtb depending essentially on the host’s immune response (3). The host immune system, when exposed to Mtb, initiates the innate immune response (4, 5) that in turn activates the adaptive immune cells (3). The adaptive immune response resulting in protective immunity against Mtb has primarily been ascribed to host resistance induced by CD4^+^ T helper type 1 (Th1) cells producing cytokines such as interferon (IFN)-γ and tumor necrosis factor (TNF) (6), in combination with the cytotoxic activity of CD8^+^ T cells (7). However, although it is clear that T cells, especially CD4^+^ T cells, are required for restricting the progression of TB, their essential contributions and their limitations are not completely defined (8, 9).

The consensus holds that a balanced immune response is necessary for a protective immunity encompassing the production of pro- and anti-inflammatory cytokines at the adequate proportion, timing and combination. Pro-inflammatory cytokines such as interleukin (IL)-1β and TNF are critical in anti-mycobacterial immunity predominantly during the early phase of Mtb infection. However, if the production of these cytokines is excessive it might result in an inefficient immune response and even considerable tissue damage (10). Thus, limiting inflammation is essential to reach a protective immune response and avoid tissue damage (11).

Upon infection with Mtb, about 90% of individuals are estimated to raise a protective immune response avoiding the progression to active TB (12). Due to our inability to discriminate the ones that eliminate Mtb from the ones that sustain the bacterial growth, individuals that are immunoreactive but with no symptoms, are referred as having latent TB. Latent TB thus represents a spectrum of outcomes ranging from clearance of the pathogen to active subclinical disease (3, 13).

Glycolipids of the Mtb cell envelope play an important role in the modulation of the innate immune response in Mtb infection (14–17). Several of these glycolipids have been shown to downmodulate the pro-inflammatory immune response. It is commonly thought that this downmodulation is unfavorable to the host. However, we have observed a hyporesponsive state of myeloid cells to glycolipids of the Mtb cell envelope in latent TB as compared to active TB (18). Since the pattern of immune response in individuals with latent TB represents a more protective profile compared to the one in patients with active TB, this suggests that downmodulation of myeloid cell functions by specific Mtb glycolipids may - in contrast to conventional thought - be beneficial for the host and protect against active TB disease.

**Figure 1:**
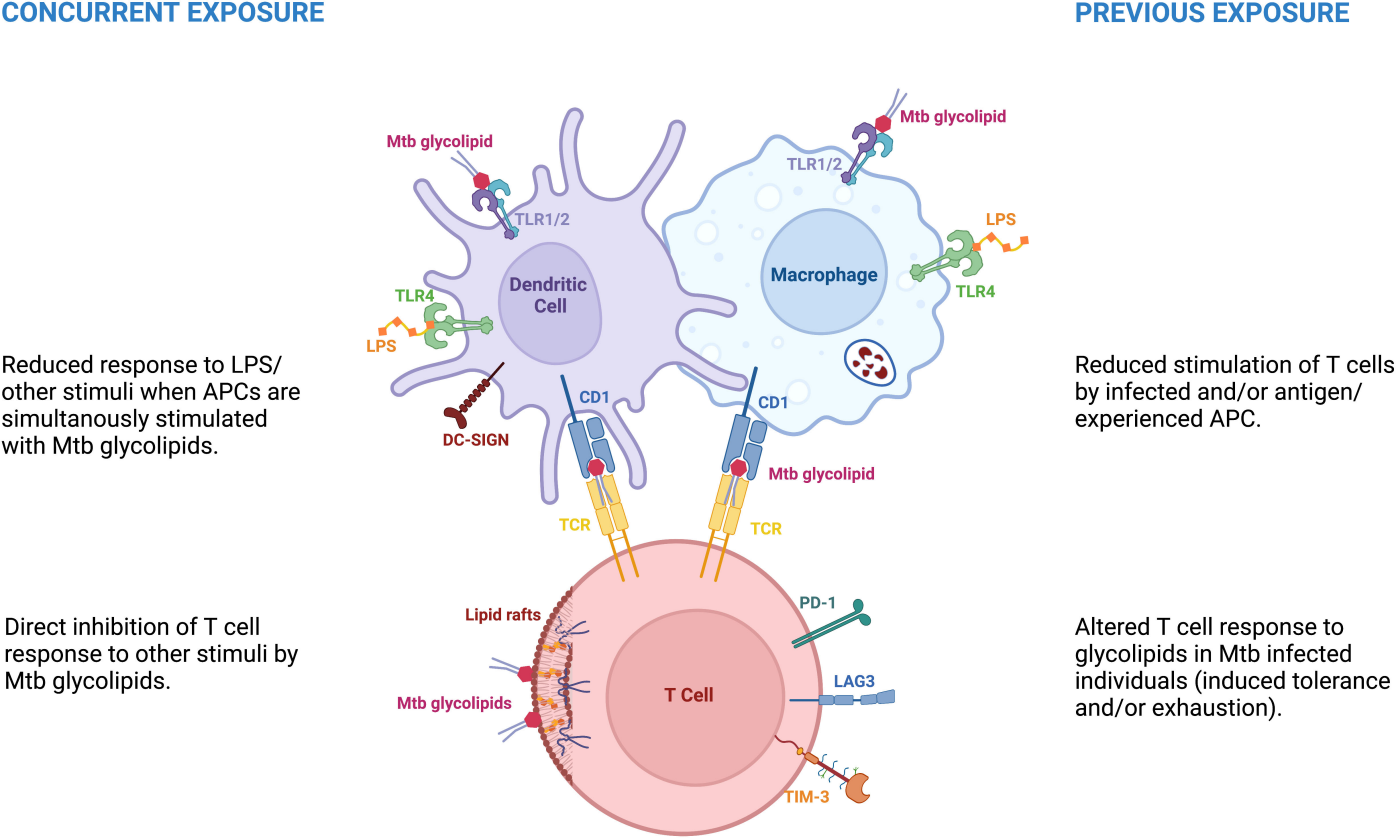
Mycobacterial glycolipids are associated with immune hyporesponse in distinct circumstances. Concurrent exposure – referred as the hyporesponse to distinct stimuli due to concurrent exposure to mycobacterial glycolipids. Previous exposure - represents the hyporesponse to glycolipids or other stimuli due to previous, repeated or continuous exposure to diverse Mtb antigens.

In this Review we discuss the effects of Mtb glycolipids in the light of their role in the immunological hyporesponsiveness following Mtb infection. We review data showing that they play an important role in the downmodulation of the immune response in individuals with Mtb infection, and in particular in those with latent TB. We further discuss various mechanisms of hyporesponsiveness induced by mycobacterial glycolipids on the innate and adaptive compartments, and their potential protective effect against active TB. On one hand the exposure of immune cells to glycolipids renders them hyporesponsive to other stimuli, such as LPS or IFN-γ. On the other hand, infection with mycobacteria may render the innate and acquired immune cells less responsive to glycolipids (**Figure 1**).

## 2 Mycobacterial glycolipids and their receptors

**Figure 2:**
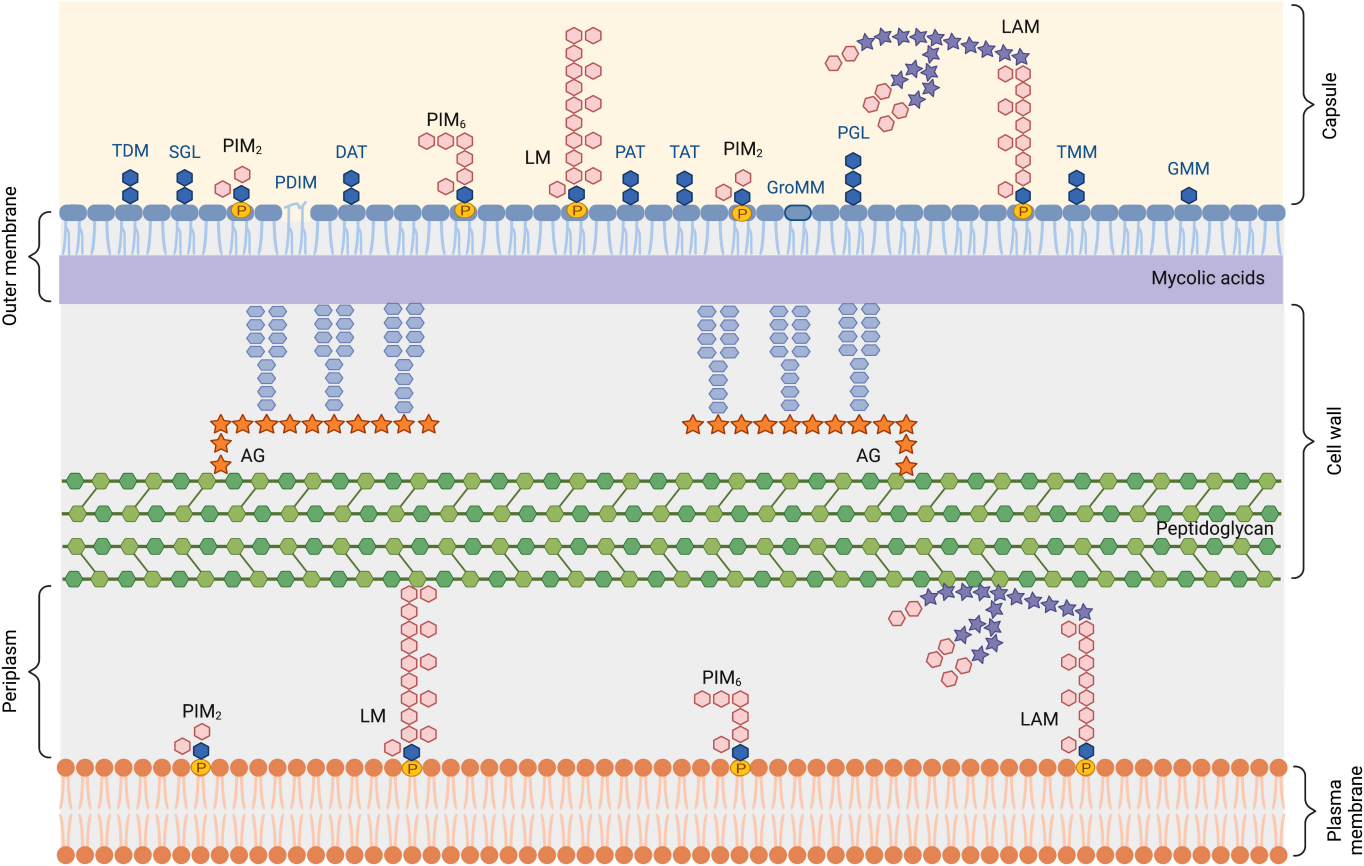
Schematic representation of the *Mycobacterium tuberculosis* cell envelope. AG, arabinogalactan; DAT, di-acyltrehalose; GMM, glucose monomycolate; GroMM, glycerol monomycolate; LAM, lipoarabinomannan; LM, lipomannan; PAT, penta-acyltrehalose; PIM, phosphatidylinositol mannoside; PDIM, phthiocerol dimycocerosate; PGL, phenolic glycolipid; SGL, sulfoglycolipid; TAT, tetra-acyltrehalose; TDM, trehalose-6,6’-dimycolate; TMM, trehalose monomycolate.

Glycolipids are important parts of the mycobacterial cell envelope (14, 19), and are also potent immunomodulatory molecules (17). The lipid-rich Mtb envelope has a unique chemical structure (19–22) (**Figure 2**), comprising: (i) a plasma or inner membrane, (ii) a cell wall core made of the peptidoglycan-arabinogalactan complex (AGP) (iii) an outer membrane (MOM), whose inner leaflet, made of mycolic acids, is covalently linked to AGP, and (iv) an outermost capsule.

The plasma membrane is composed of phospholipids and phosphatidylinositol (PI)-derived glycolipids, namely phosphatidylinositol mannosides (PIMs), lipomannan (LM) and lipoarabinomannan (LAM). The inner leaflet of the outer membrane is made of long-chain mycolic acids that esterify the terminal arabinose residues of AGP. The outer leaflet is composed of a large diversity of non-covalently attached (glyco)lipids, that can be classified in different families (23), i.e. PI-derived glycolipids, mycolic acids and conjugates, multimethyl-branched and polyunsaturated fatty acid esters of trehalose, and mycocerosate-containing lipids, detailed in the following paragraphs.

The capsule is mostly made of proteins and polysaccharides including α-glucan, arabinomannan (AM) and mannan, that correspond to lipid-free forms of LAM and LM (24, 25).

### 2.1 *Mycobacterium tuberculosis* glycolipid structural diversity

#### 2.1.1 Phosphatidylinositol (PI)-derived glycolipids

LAM, LM and PIM share a common lipid anchor, namely Mannosyl-Phosphatidyl-*myo*-Inositol (MPI), which contains two acylation sites on the glycerol unit and two additional potential ones, on the *myo*-inositol unit and on the mannose unit linked at *O*-2 of the *myo*-inositol (22, 26). Palmitic and tuberculostearic acids are the major fatty acids. The MPI anchor can be glycosylated by one to five mannosyl units, yielding PIM, phosphatidylinositol dimannosides (PIM_2_) and hexamannosides (PIM_6_) being the most abundant glyco-forms. LM is made by polymannosylation of tri-acylated PIM_2_ (bearing a fatty acid on the mannose unit in addition to both positions of glycerol). LAM corresponds to LM with an attached arabinan domain. In Mtb, the non-reducing termini of the arabinosyl side chains of LAM are modified by mono-, di- or tri-mannoside units, called mannose caps (ManLAM). In the present review, unless otherwise stated, the term LAM will refer to ManLAM.

#### 2.1.2 Mycolic acids and conjugates

Mycolic acids (MAs) are 3-hydroxy-2-alkyl-branched long-chain (60-90 carbon atoms) fatty acids bearing different chemical functions, such as double bonds, cyclopropanes, oxygenated functions at two distinct positions along the meromycolic chain (27). MAs are found either covalently attached to the AGP to form the inner leaflet of the MOM, or as free lipid conjugates (esters) participating in the composition of the outer leaflet of the MOM. Esters of mycolic acids include trehalose-6,6’-dimycolate (TDM; also known as cord factor because of its association with the cording morphology of Mtb), trehalose monomycolate (TMM), glucose monomycolate (GMM) or glycerol monomycolate (GroMMs) (23).

#### 2.1.3 Multimethyl-branched and polyunsaturated fatty acid esters of trehalose

In addition to TDM and TMM mentioned above, trehalose serves as a scaffold for esterification by a wide range of fatty acids other than mycolic acids (21, 23). Di- (DAT), Tri- (TAT), and Penta- (PAT) acyl trehaloses represent a second group of lipids of this family. Trehalose is acylated with a simple palmitic or stearic acid at position 2 of the sugar core and at other positions with methyl-branched mycolipenic, mycolipodienoic, mycolipanolic or mycosanoic acids. Sulfoglycolipids constitute a third group of glycolipids in this family. Sulfoglycolipids are trehalose 2’-sulfates acylated at position 2 by a straight-chain palmitic or stearic acid, 3, 6, and 6’ with one to three phthioceranic or hydroxyphthioceranic acids.

#### 2.1.4 Mycocerosate-containing lipids

They comprise phthiocerol dimycocerosates (PDIM) and phenolic glycolipids (23). PDIM are characterized by a long-chain β-diol esterified by two polymethyl-branched fatty acids, named mycocerosic acids. In phenolic glycolipids, produced by a few Mtb strains only, this lipid is ω-terminated by an aromatic nucleus and glycosylated by a trisaccharide.

### 2.2 *Mycobacterium tuberculosis* glycolipids are ligands of Toll-like Receptor 2 and C-type lectins

Most of the Mtb cell envelope lipids described above have been demonstrated to possess immunomodulatory properties which rely on their ability to (i) act as ligands (either agonists or antagonists) of pattern recognition receptors (PRRs), mainly Toll-like receptor 2 (TLR2) and C-type lectins, (ii) mask pathogen-associated molecular patterns (PAMPs) at the surface of the Mtb cell envelope and prevent their interaction with PRRs, (iii) directly insert into the host cell membranes and thereby interfere with cell functions, and/or (iv) to act as antigens presented by CD1 molecules to unconventional T cells.

#### 2.2.1 TLR2

TLRs are a family of PRRs expressed on innate immune cells such as macrophages and dendritic cells (DCs) as well as neutrophils (28), and that detect a wide range of PAMPs (29). They trigger NF-κB-dependent and IFN regulatory factor (IRF)-dependent signaling pathways. Of all TLRs, TLR2 is the one that binds to the structurally broadest range of PAMPs, including lipoproteins and lipopeptides, lipoteichoic acid, lipoglycans and glycolipids. All the Mtb lipid families described above comprise ligands of TLR2.

Among all the TLR2 agonists, the strongest is LM followed by PIM_6_, while LAM and PIM_2_ are much weaker agonists (30–32). Indeed, TLR2 agonist activity of PI-based glycolipids increases with the length of the mannan chain, while it is thought that the bulky arabinan domain of LAM prevents an efficient interaction with the receptor (30, 33). Only tri- and tetra-acylated forms of PI-based glycolipids are recognized by TLR2, in cooperation with TLR1 and CD14 (34). TDM, DAT and TAT also induce TLR2-dependent immune responses, while requiring different accessory receptors (35, 36).

Sulfoglycolipids are also ligands of TLR2. In contrast to previous lipids, they are competitive antagonists of TLR2, blocking NF-κB activation and subsequent cytokine production or costimulatory molecule expression (37). Both tetra- and di-acylated sulfoglycolipids are TLR2 antagonists. Fatty acids and the sulfate group are required for sulfoglycolipids to compete for the binding of TLR2 agonists, lipoproteins or LM (37).

Phenolic glycolipids have also been described to inhibit TLR2 activation, by a yet unclear mechanism that seems to involve binding of the saccharidic part of the molecule to TLR2 (38).

Finally, PDIM have been proposed to mask in part TLR2 agonists at the bacterial cell surface, restricting their accessibility to the receptor, thereby limiting Mtb recognition (39).

#### 2.2.2 C-type lectin receptors

C-type lectins (CLRs) are a family of receptors that characteristically interact with terminal mono- or oligosaccharide units of large carbohydrates in a calcium-dependent manner (40). CLRs such as the mannose receptor, Dendritic Cell-Specific Intercellular adhesion molecule-3-Grabbing Non-integrin (DC-SIGN), Macrophage inducible Ca^2+^-dependent lectin receptor (Mincle) and Dectin-2, are PRRs that specifically bind carbohydrates present on pathogens (16, 40). Through interaction with their specific PAMPs they induce signaling pathways, either by directly inducing immune-related gene expression or by modulating TLR signaling (16, 41).

DC-SIGN is expressed on the surface of DCs. It recognizes and binds to high-mannose-containing glycans, a class of PAMPs found on various microorganisms including Mtb, as well as Lewis-type structures with fucose units, and glucans (42, 43). The immunological outcome of DC-SIGN triggering varies depending on the pathogen involved (44), and the structure of the molecule bound by DC-SIGN determines DC-SIGN-mediated uptake and trafficking (45).

Purified LAM (43, 46), LM (47) and PIM_6_, but not PIM_2_ (48) are ligands of DC-SIGN. Binding is mediated *via* terminal mannosyl units. Other Mtb non-lipid ligands of DC-SIGN include AM, mannan, mannoproteins (such as Apa or the 19 kDa lipoprotein) (47), as well as α-glucan (49). However, the molecular basis of Mtb recognition by DC-SIGN are not clearly understood. Indeed, whereas most of the ligands mentioned above are produced by all mycobacterial species, DC-SIGN selectively recognizes Mtb complex species (47). In addition, the study of several Mtb complex mutants indicate that none of these ligands is dominant or essential, suggesting functional redundancy (48, 50).

The mannose receptor is expressed primarily by macrophages and DCs. In macrophages, engagement of the mannose receptor by LAM during the phagocytic process directs Mtb to its initial phagosomal niche, thereby enhancing Mtb survival (51). As for DC-SIGN, LM and PIM_6_, but not PIM_2_ bind the mannose receptor *via* their terminal mannosyl units (52, 53). Binding is modulated by acylation degree of the molecules, most probably as a result of an impact on the clustering effect that is important for multivalent high affinity binding to C-type lectins (54).

The Dectin-2 family/cluster encompasses several C-type lectins that recognize Mtb glycolipids, notably Dectin-2, Mincle, and DCAR (16, 55). Dectin-2 is expressed by both DCs and macrophages (56). In murine bone-marrow-derived dendritic cells (BMDCs), Dectin-2 triggers the expression of pro-inflammatory cytokines, such as TNF, IL-6, and IL-12p40, as well as IL-10 (56). It also enhances the functions of antigen-presenting cells (APCs) to promote T cell production of IL-17 (56). Dectin-2 has recently been found to play a role in the modulation of early hyper- and late hypo-immunoreactivity in sepsis by a cell wall N-glycan of *Candida albicans* (57), indicating that Dectin-2 may play a role in immunosuppression like other C-type lectins including DC-SIGN. Dectin-2 is a receptor for LAM (56), which is the sole Mtb ligand of Dectin-2 as demonstrated by the use of isogenic mutants (58). Dimannoside caps and multivalent interaction are required for ligand binding to and signaling *via* Dectin-2 (58).

Mincle is strongly expressed on macrophages but also on DCs. It binds glycolipids containing glucose or mannose (59). Ligand binding to Mincle leads to phosphorylation of the immunoreceptor tyrosine activation motif (ITAM) of the FcRγ chain leading to activation of NF-κB *via* Syk-Card9–Bcl10–Malt1 signaling (60).

Both human and mouse Mincle bind a large number of Mtb glycolipids (59), TDM, TMM and GlcMM being the strongest agonists (36). GroMM binds to human Mincle only (61), although with a lower efficiency than the mycolic acid esters mentioned above. Free mycolic acids are also recognized by the receptors (36). DAT and TAT are also agonists of both human and mouse Mincle (36).

Dendritic cell immunoactivating receptor (DCAR) is found on tissue inflammatory cells derived from circulating monocytes in mice. No homolog has been found in humans so far. Like Mincle, DCAR induces signaling through the ITAM motif of FcRγ (62), promoting Th1 responses during mycobacterial Infection (63). DCAR recognizes PIMs by binding both their saccharidic and lipid moieties (64).

The DAP12-associated triggering receptor expressed on macrophage 2 (TREM2) is expressed on various myeloid cells, including macrophages. It preferentially recognizes free mycolic acids, rather than mycolic acid esters that are sensed by Mincle (65). Triggering of TREM2 signaling by mycolic acids inhibits Mincle-induced macrophage activation (65).

In summary, many glycolipids in the Mtb envelope are potent modulators of the immune response through signaling *via* receptors such as TLR2, CLRs, and TREM2 on monocytes/macrophages and DCs. Some induce strong pro-inflammatory responses, *via* TLR2 or CLRs, the most prominent example being the action of TDM *via* Mincle. Others act through receptors such as DC-SIGN or Trem2 to downmodulate a pro-inflammatory response. However, it is important to note that a given glycolipid, for instance LAM, may be recognized by several receptors, triggering different responses (pro- vs anti-inflammatory) according to the receptor engaged and potential regulation of their signaling pathways.

## 3 Downmodulation of myeloid cell function by Mtb glycolipids

Infection with Mtb is known to activate certain myeloid cell functions while inhibiting others. The ability of Mtb glycolipids to inhibit specific myeloid cell functions has been investigated extensively using diverse *in vitro* myeloid cell models (**Table 1**).

**Table 1:** In vitro effect of Mtb glycolipids on the response of monocytes/macrophages and DCs to other stimuli.

Cell type	Primary stimulation or simultaneous primary/secondary stimulation		Secondary stimulation		Cytokine production and cell maturation	Modulation effect	Ref
	Stimulant	Incubation time	Stimulant	Incubation time			
**Monocytes/Macrophages**							
Human monocytes and human monocytic cell line TPH-1	LAM (2 μg/ml)	16 h	LPS (1 μg/ml)	15 min	TNF ↓ IL-12 ↓ compared to no LAM	Downmodulation of LPS effect induced by LAM	(66)
Mouse BMD macrophages + IFN-γ	LAM from BCG (10 μg/mL) + LPS (100 ng/mL)	24 h	NA	NA	TNF ↓ IL-12 ↓ compared to no LAM	Downmodulation of LPS effect induced by LAM	(67)
Mouse macrophage cell line RAW264.7	LAM (5-20 μg/mL)	16 h	LPS (1 μg/mL)	24 h	IL-12 ↓ compared to no LAM	Downmodulation of LPS effect induced by LAM	(68)
Human monocytes	LAM (1 μg/ml)	24, 48, 72, 96, 120 h (followed by 7 days resting)	LPS (1 μg/ml)	24 h	TNF ↓ after 72 h LAM IFN-γ →	Downmodulation of LPS effect induced by LAM	(69, 70)
Mouse BMD macrophages	di-acylated LM from BCG (3 μg/ml) + LPS (100 ng/ml)	24 h	NA	NA	TNF ↓ compared to no LM	Downmodulation of LPS effect induced by LM	(71)
Mouse BMD macrophages TLR-2 deficient and wildtype	PIM2 and PIM6 (6.7 μg/ml)	30 min	LPS (100 ng/ml)	24 h	TNF ↓ IL-12/IL-23 p40 ↓ IL-6 ↓ IL-10 ↓ compared to no PIM	Downmodulation of LPS effect induced by PIM	(72)
BMD macrophages	Phenolic glycolipid from W-Beijing Mtb (2 μg/ml) + apolar lipids (1 μg/ml)	24 h	NA	NA	TNF ↓ IL-6 ↓ compared to no PGL	Downmodulation of apolar lipid effect induced by PGL	(73)
Human monocyte cell line THP-1	SGLs (dose-response) + Mtb lipoproteins (0.5 µg/ml)	16 h	NA	NA	NF-κB ↓ IL-8 ↓ CD40 expression ↓ compared to no SGLs	Downmodulation of Mtb lipoprotein effect induced by SGLs	(37)
Immature DCs 5 days with GM-CSF + IL-4	LAM from BCG (10-50 μg/mL) + LPS (2 ng/ml)	48 h	NA	NA	IL-12 ↓ in a dose dependent manner compared to no LAM	Downmodulation of LPS effect induced by LAM	(74)
**Dendritic Cells**
Immature DCs 5 days with GM-CSF + IL-4	LAM from BCG (10 μg/mL) + LPS (20 ng/ml)	18 h	NA	NA	TNF ↓ compared to no LAM	Downmodulation of LPS effect induced by LAM	(75)
Immature DCs 24 h with GM-CSF + IL-4	LAM (15 μg/mL) + LPS (10 ug/mL) + GMCSF + IL-4	18 h	NA	NA	IL-10 ↑ ↓ maturation compared to no LAM	Downmodulation of LPS induced maturation by LAM and increase of IL-10	(42)
Immature DCs 6-7 days with GMCSF + IL-4	LAM (10 µg/ml) + LPS (10 ng/ml)	24 h	NA	NA	IL-10 ↑ compared to no LAM	Increase of IL-10 by LAM in LPS treated cells	(76)
Immature DCs 6-7 days with GMCSF + IL-4	LAM (10 µg/ml) + LPS (10 ng/ml)	24 h	NA	NA	IL-6 ↑ IL-10 ↑ IL-12p35 ↑ IL12p40 ↑ compared to no LAM	Upregulation of LPS effect by LAM	(44)
Immature DCs 6 days with GMCSF + IL-4	LAM (10 µg/ml) + LPS (100 ng/ml)	12 h	NA	NA	TNF ↑ IL-6 ↑ IL-12 ↑ activation/maturation compared to no LAM	Upregulation of LPS effect by LAM	(77)
Immature DCs 6 days with GMCSF + IL-4	PIM (5 µg/ml) + LPS (100 ng/ml)	12 h	NA	NA	Dose dependent TNF ↓ IL-6 ↓ IL-12p40 ↓ compared to no PIM	Downmodulation of LPS effect by PIM	(77)

BMD, Bone-marrow-derived; BMDC, Bone-marrow-derived dendritic cell; DAT, Di-O-acyl- trehalose; LAM, Lipoarabinomannan; LM, Lipomannan; LPS, Lipopolysaccharide; MD, Monocyte derived; MDDC, Monocyte-derived dendritic cell; PIM, Phosphatidylinositol mannoside; SGL, Sulfoglycolipid.

Symbols used: ↑ Increased production; ↓ Decreased production; → No difference in relation to Mtb glycolipid; NA, Not applicable.

In monocytes/macrophages, LAM has been shown to downmodulate the production of IL-12 and/or of TNF induced by lipopolysaccharide (LPS) (66–69). *In vitro* work with DCs also revealed that LAM decreases the response to LPS by these cells (42, 74, 78–80) by suppressing DC maturation (42, 80) and reducing IL-12 secretion (74). Geijtenbeek et al. found that LAM binding to DC-SIGN prevents mycobacteria- or LPS-induced DC maturation (42), and TLR signaling is modulated *via* the involvement of an intracellular signalosome (44, 76). Nigou et al. showed that in human DCs LAM suppressed IL-12 production induced by LPS by engagement of the mannose receptor. As for DC-SIGN, the authors suggest that engagement of the mannose receptor by LAM delivers a negative signal that interferes with the LPS-induced positive signals delivered by TLR4 (74).

LAM’s effect on IL-10 production by DCs, when receiving a simultaneous activation signal, like LPS, has been investigated by several groups. In most situations the authors reported an increased production of IL-10 (42, 80). This effect was ascribed to its binding to C-type lectins such as the mannose receptor and DC-SIGN (42, 44, 76). Accordingly, α-glucan, a polysaccharide from the Mtb capsule induces a DC-SIGN-dependent production of IL-10 by LPS-activated monocyte-derived DCs (49) (**Table 1**).

In contrast to the induction of hyporesponsiveness described above, Gringhuis et al. later reported that LAM binding to DC-SIGN enhanced both pro-inflammatory immune responses and IL-10 production induced by LPS (44). Consistent with this, Mazurek et al. found that LAM, but not PIM, from Mtb H37Rv and bacillus Calmette-Guérin (BCG), when given simultaneously with LPS, promoted LPS-induced DC maturation and pro-inflammatory cytokine production (77).

Experiments on the effects of LAM alone on cytokine production and DC maturation generated also some controversial results; while Mazurek et al. reported that LAM stimulated the production of TNF and IL-12p40 and drove DCs maturation/activation (77), others observed no effect of LAM alone in stimulating DCs (44). In neutrophils, LAM induced the production of cytokines (IL-1Ra, IL-6, and IL-8) in a TLR2/1-dependent manner, but did not elicit p38 MAPK phosphorylation (81), suggesting a different TLR2/1 signaling pathway than in monocytes/DCs.

The reasons for these different observations remain unclear. Comparing the protocols, the authors all use the same cell type (human monocyte-derived DCs), although the cells may differ in activation states (i.e. expression of receptors and activity of signaling pathways). The fact that LAM alone upregulated pro-inflammatory cytokines as reported by Mazurek et al. (77) could indicate that in this study the upregulation of the LPS-induced cytokines by LAM does not go through the DC-SIGN/TLR signaling pathway, but e.g. *via* Dectin-2, which, in contrast to the TLRs, activates macrophages and DCs through a Syk-Card9 dependent signaling pathway (82). Indeed, it was later reported that LAM binding to Dectin-2 induces both pro- and anti-inflammatory cytokine production (56). Other contributing factors, related to the potential induction of immune tolerance (see further under the section "3.3 *Primary stimulants may induce tolerance or trained immunity in vitro depending on experimental protocol"*) could be differences in the dose of LPS (higher in Gringhuis et al. [10 ng/ml] and Mazurek et al. [100 ng/ml] compared to Nigou et al. [2 ng/ml]), the purity of LPS, and/or incubation time. These factors are of importance in the induction of tolerance to LPS. Chavez-Galan et al. (69) reported that a minimum of 74 h of stimulation with LAM was necessary to downmodulate LPS-induced TNF production, whereas other studies observed a downmodulation after 18 h (75) or 48 h (74).

*LM* (33, 34, 67) and *PIM* (83) are TLR2 agonists that induce the production of pro-inflammatory cytokines in macrophages and the formation of granuloma (84, 85). Yet, both LM and PIM can also inhibit the LPS-induced TLR4-mediated pro-inflammatory cytokine production (TNF, IL-6, IL-12) by macrophages (67, 71, 72, 86) or human monocyte-derived DCs (77). In addition, prolonged TLR2 signaling induced by Mtb lipoproteins and, potentially, by other TLR2 agonists such as LM and PIM (87) results in inhibition of MHC class II molecule expression and antigen presentation by Mtb-infected macrophages (88).

*Sulfoglycolipids* inhibit NF-κB activation and subsequent cytokine production (induced by Mtb lipoproteins) by acting as competitive antagonists of TLR2 in human macrophages (37), thereby inhibiting the recognition of Mtb by TLR2.

*Phenolic glycolipids* downmodulate the host innate immune response (89). Phenolic glycolipids produced by a subset of Mtb isolates decrease the production of pro-inflammatory cytokines induced by apolar glycolipids in Mtb-infected monocytes or monocyte-derived macrophages in a dose-dependent manner (73) (**Table 1**). In agreement, infection with a modified strain of Mtb that does not express phenolic glycolipids was found to correlate with an increased release of pro-inflammatory cytokines *in vitro* (73). The trisaccharide domain of the phenolic glycolipids from Mtb and *M. leprae* share the capacity to inhibit TLR2-triggered NF-κB activation, and thus the production of pro-inflammatory cytokines (38).

*TDM* binding to Mincle activates macrophages and DCs to produce pro-inflammatory cytokines and nitric oxide (36, 59, 60) and triggers the formation of granuloma (59, 90). Yet, TDM exerts delayed inhibition of IFN-γ-induced gene expression, including pattern recognition receptors, MHC class II genes, and IFN-γ-induced GTPases, with antimicrobial function (91). Moreover, beads coated with TDM induce a Mincle-dependent anti-inflammatory IL-10 response that counter-regulates IL-12 production in macrophages (92). In addition, it interferes with FcγR-mediated phagosome maturation through Mincle, SHP-1 and FcγRIIB signaling (93).

*Mycolic acids* induce a TREM2-dependant pathway that leads to recruitment of inducible nitric oxide synthase (iNOS)-negative mycobacterium-permissive macrophages and counteracts Mincle-FcRγ-CARD9-mediated inflammatory cytokine production (65). TREM2 is responsible for blocking the production of TNF, IL-1β, and Reactive oxygen species (ROS), while enhancing the production of IFN-β and IL-10 (94).

*DAT* has been shown to induce tolerance in bone marrow-derived (BMD) murine DCs that were activated with delipidated BCG cell wall and TLR agonists, leading to decreased antigen presentation and less production of IL-12 and increased levels of IL-10 (95) (**Table 1**). DAT was previously shown to activate both human and mouse Mincle and induce cytokine production (36). Whether DAT-induced tolerance is mediated by Mincle remains to be investigated.

### 3.1 Mechanisms of induction of hyporesponsiveness by Mtb glycolipids

**Figure 3:**
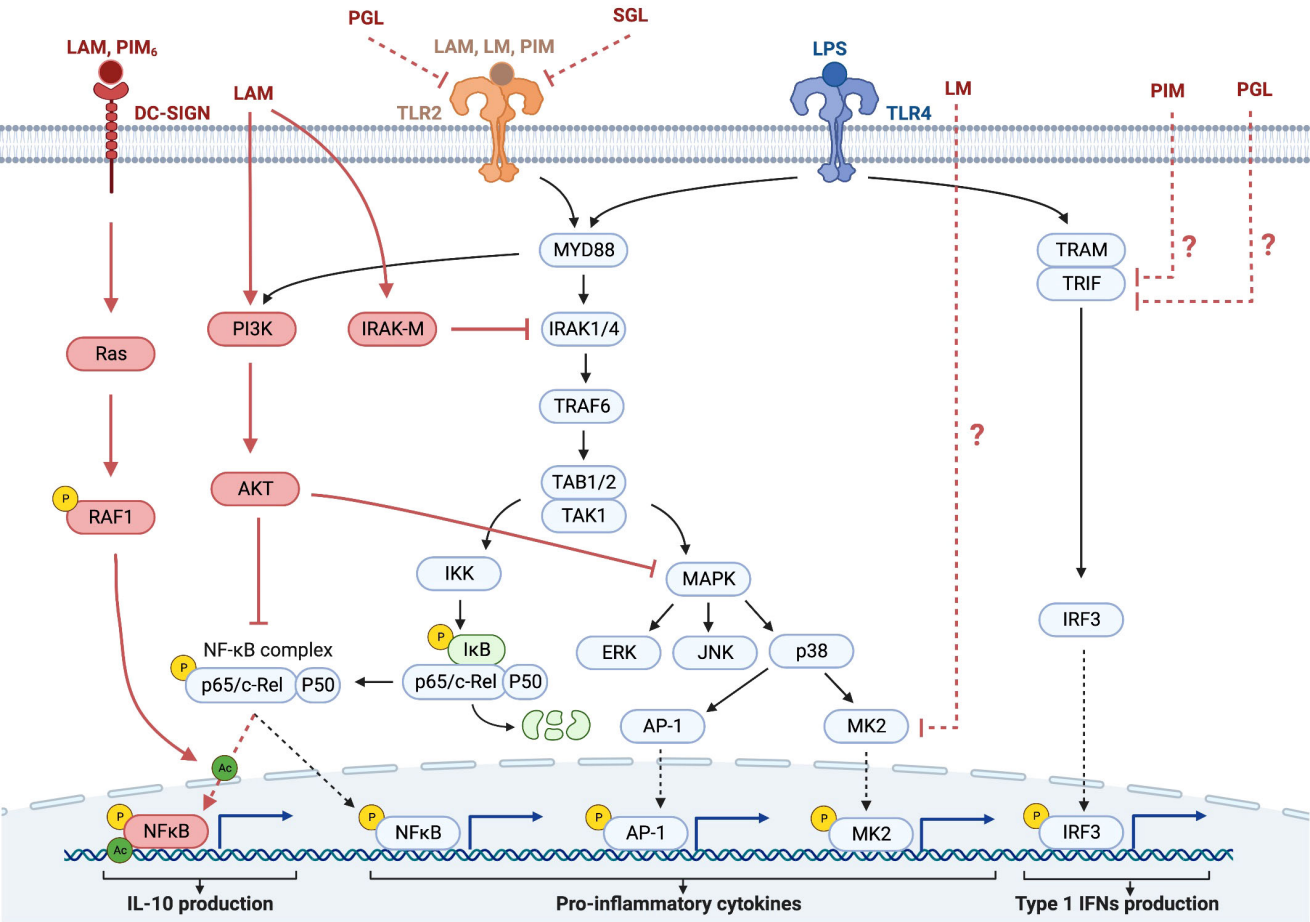
Some of the signaling pathways in the context of hyporesponsiveness in monocytes/macrophages and DCs. The TLR signaling pathways induced by PAMPs, such as LPS and Mtb glycolipids activate NF-κB and MAPK cascades in macrophages and DCs, leading to the production of pro-inflammatory cytokines. Most TLRs bind the adaptor protein MyD88, initiating signaling through the serine/threonine kinase IRAK, which then associates with the adaptor protein TRAF6. After the activation of the IKK complex, IκB, an inhibitor of NF-κB, becomes phosphorylated and then degraded. This leads to the activation of NF-κB and its translocation to the nucleus with subsequent production of immunostimulatory cytokines and DC maturation. Interaction with DC-SIGN, which is mainly expressed on the surface of DCs, activates the small GTPase, Ras, leading to the activation of NF-κB by phosphorylation of the p65 subunit at Ser276 and its subsequent acetylation, thereby enhancing the production of the immunosuppressive cytokine IL-10 (44, 76, 99, 100). The negative regulator of TLR signaling, IRAK-M, is induced in macrophages and DCs in response to the first activation of the TLRs and functions as a negative regulator in the second or continuous stimulation by TLR agonists. The IRAK family includes two active kinases, IRAK and IRAK-4, and two inactive kinases, IRAK-2 and IRAK-M. IRAK-M inhibits further downstream activation of NF-κB by preventing the dissociation of IRAK and IRAK-4 from MyD88 and the formation of IRAK-TRAF6 complexes (101). Another negative regulator of TLR signaling is PI3K, which is constitutively expressed in innate immune cells, such as DCs and macrophages. Unlike IRAK-M, PI3K functions at the early phase of TLR signaling in response to the first encounter with the pathogens. PI3K activates AKT (also known as PKB), which inhibits both NF-κB and the MAPK pathway, leading to reduced inflammatory cytokine production (102). In addition to the activation of NF-κB, TLR ligation also activates the MAPK pathway. MK2, is a kinase that is downstream of p38 and regulates the synthesis of pro-inflammatory cytokines. Downmodulation of MK2 therefore results in reduced cytokine production. TLR4 ligation can in addition to MyD88 also signal *via* TRIF to IRF3 leading to production of type 1 IFNs (103). DCs, dendritic cells; TLR, Toll-like receptor; PAMP, pathogen associated molecular patterns; LPS, lipopolysaccharide; MAPK, mitogen-activated protein kinase; IRAK, iIL-1 receptor-associated kinase; TRAF6, TNF-receptor-associated factor 6; IKK, inhibitor of NF-κB kinase; LAM, lipoarabinomannan; DC-SIGN, DC-specific intracellular adhesion molecule-grabbing non- integrin; IRAK-M, IL-1 associated kinase-M; PGL, phenolic glycolipid; SGL, sulfoglycolipid.

Early studies of hyporesponsiveness mainly explored the direct role of Mtb glycolipids in the immune response to stimulation of myeloid cells (**Table 1**), where transcription of genes takes place at the time of stimulation in response to a ligand directly acting on the cell (66, 72, 96). In these studies, LPS was frequently used as the trigger of macrophage-mediated inflammation. Since the main receptor for LPS is TLR4, this receptor has been extensively explored in this context (97, 98). Two major signaling pathways have been described in the activation of macrophages by LPS *via* TLR4 (**Figure 3**). These pathways involve either the adaptor protein Myeloid differentiation factor 88 (MyD88) or the Toll/IL-1-receptor (TIR)-domain-containing adaptor-inducing IFN-β (TRIF) (104). Both pathways converge in their activation of the transcription factor NF-κB. Prolonged activation of TLR4 can lead to reduced activation of downstream kinases, such as IL-1 receptor-associated kinase (IRAK) and MAPKs. Most TLRs, including TLR2 and TLR4, are activated *via* the MyD88-dependent signaling pathway, while TRIF is involved in TLR4 but not TLR2 signaling.

A major mechanism involved in the hyporesponsiveness induced by Mtb glycolipids is the inhibition of the transcription factor NF-κB, which has been shown for LAM (72), PIM (72), phenolic glycolipids (38), sulfoglycolipids (49), and α-glucans (49). The signaling pathways leading to the downmodulation of NF-κB by Mtb glycolipids in most cases remain to be explored. In macrophages, LAM downmodulates the IL-12 production induced by LPS by interfering with MyD88, IRAK, and TNF receptor-associated factor (TRAF) complexes (68) (**Figure 3**) through IRAK-TRAF6 interaction, that attenuates nuclear translocation and DNA binding of c-Rel and p50. LAM exerts these effects by inducing the expression of IRAK-M, a negative regulator of TLR signaling (105). Knockdown of IRAK-M expression by RNA interference reinstated LPS-induced IL-12 production by LAM-pretreated cells (68) (**Figure 3**). LAM has also been shown to activate PI3K and Akt, indicating another pathway for LAM to downmodulate NF-κB (**Figure 3**).

As discussed previously the effect of LAM on monocytes/macrophages and DCs has been addressed in several instances. In DCs the downmodulation of IL-10 production by LAM occurs through binding to C-type lectins such as the mannose receptor (74) and DC-SIGN (76). Gringhuis et al. showed that the effect of LAM on LPS stimulation, through DC-SIGN, triggers a molecular signaling pathway that modulates TLR signaling at the level of NF-κB, by activating the serine and threonine kinase Raf-1, which subsequently leads to acetylation of the NF-κB subunit p65, but only after TLR-induced activation of NF-κB. Acetylation of p65 both prolonged and increased *IL10* transcription (76). The same group earlier reported that LAM inhibited the maturation of DCs, which was reversed by antibodies against DC-SIGN (42).

Interestingly, Quesniaux et al. found, using murine BMD macrophages, that the inhibitory effect of BCG LM on LPS-induced IL-12p40 production was independent of TLR2 and MyD88, suggesting that the inhibitory effect was independent of TLR-signaling (67).

Rajaram et al. found that TNF biosynthesis was blocked in human macrophages by LM by regulating macrophage MAPK-activated protein kinase 2 (MK2) and microRNA miR-125b (106) (**Figure 3**). Doz et al. found that in murine BMD macrophages, PIM inhibited LPS-induced functional responses (*in vitro* as well as *in vivo*), through both the MyD88 and TRIF signaling pathways (72) (**Figure 3**). PIM inhibited TLR4 and MyD88-mediated release of pro-inflammatory cytokines and of IL-10. PIM also reduced the MyD88-independent, TRIF-mediated expression of co-stimulatory receptors and inhibited LPS/TLR4-induced NF-κB translocation (72). Phenolic glycolipids from BCG selectively disable TRIF-dependent TLR4 signaling in macrophages (107).

Results from *in vitro* studies on the hyporesponse to LPS and other stimuli, induced by co-stimulation with LAM and PIM are in line with studies of cells from individuals with Mtb infection (**Table 2**). Whole blood cells from patients with active TB and contacts stimulated with LAM showed reduced production of TNF and IFN-γ compared to healthy controls, and lower production of IL-10 in contacts compared to patients with TB (108). We found that stimulation of peripheral blood mononuclear cells (PBMCs) with LAM and PIM induced a greater response in cells from healthy controls compared with cells from individuals with active or latent TB. The cytokines IL-1a, IL-10 and IL-18, and VEGF were secreted at lower levels from PBMCs from those with active and latent TB compared with healthy controls, indicating a hyporesponsive state in active and latent TB (18). For myeloid cells, a reduction of cytokine^-^producing cells was observed in individuals with latent TB upon stimulation with LAM and PIM. This effect was mainly observed for IL-10 and IL-6 (18).

**Table 2:** Effect of *ex vivo* stimulation of immune cells with Mtb antigens/glycolipids in clinical manifestations of Mtb infection.

Cell type	Stimulant	Active TB	Latent TB(*or contacts)	Additional information	Ref
Whole blood	LAM	TNF ↓ IFN-γ ↓ IL-10 ↑	TNF ↓ IFN-γ ↓ IL-10 ↓ *	Individuals included as contacts were living in the same house as smear positive pulmonary TB patients for 1 week or more.	(108)
PBMC	LAM	→	→		(18)
PBMC	PIM	IL-1α ↓ IL-10 ↓ IL-18 ↓	IL-1α ↓ IL-10 ↓ IL-18 ↓		(18)
Myeloid cells	LAM	IL-10 ↓ IL-6 ↓	IL-10 ↓ IL-6 ↓		(18)
Myeloid cells	PIM	→	IL-10 ↓ IL-6 ↓		(18)
T cells	PIM (PIM2/PIM6)	IL-10 ↓ IL-17A ↓ IL-6 ↓	IL-10 ↓ TNF ↓ IL-17A ↑ IL-6 ↑	Lack of expansion of naïve CD8^+^ T cells expressing GMCSF in latent TB vs healthy controls.	(18)
CD1^+^CD4^+^ T cells	Mtb lipids	IFN-γ → Proliferation →	IFN-γ ↑ Proliferation ↑	IFN-γ and proliferation increased in latent TB but not active TB.	(109)
CD1^+^CD8^+^ T cells	LAM	IFN-γ → (post-TB patients)	IFN-γ ↑		(110)
CD1^+^ T cells	Glycerol monomycolate/ mycolic acids	IFN-γ →	IFN-γ ↑		(111)
CD1^+^ T cells	Sulfoglycolipids	IFN-γ ↑	IFN-γ ↑	No difference between active TB and latent TB.	(112)

LAM, Lipoarabinomannan; PIM, Phosphatidylinositol mannoside.

Symbols used: ↑ Increased production; ↓ Decreased production; → No difference detected; all in relation to healthy controls.

### 3.2 Myeloid cell tolerance and trained immunity

**Figure 4:**
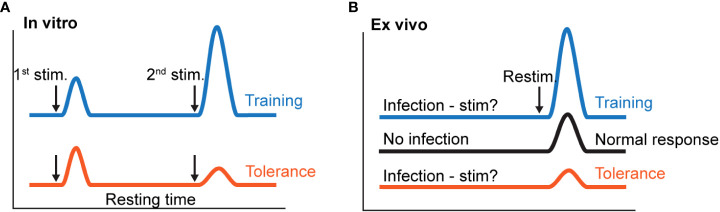
Schematic presentation of induction of innate training vs tolerance. **(A)** Innate training or tolerance can be achieved *in vitro* by using different times of primary stimulation and resting of immune cells, different concentrations of the stimuli, and different secondary stimuli. **(B)** *In vivo* innate training and tolerance can potentially be achieved by infection (primary stimulation) for varying times and doses and expressed *ex vivo* upon secondary stimulation of immune cells from the infected individual.

More recently, the hyporesponsiveness described above is discussed in the context of innate immune memory. Epigenetic changes of innate immune cells are central to this process and may result in a long-term adaptation of innate immune cells leading either to enhanced responsiveness (trained immunity) or a weakened response (innate tolerance) to a subsequent challenge (113–115) (**Figure 4A**). The magnitude and duration of stimulation induce specific adaptations in innate immune cells that either enhance or attenuate the immune responses (**Figure 4B**).

The BCG vaccine was the first stimulus to be identified to induce trained immunity, and its effect has been extensively studied both *in vivo* (113) and *in vitro* (116, 117). Monocytes/macrophages infected with BCG *in vitro* develop trained immunity, with increased cytokine production upon a secondary stimulus (116, 117).

Although the phenomenon of trained immunity has been extensively studied, innate immune tolerance has received less attention in the context of infectious diseases (97). Innate tolerance as a concept has been observed for many decades (118), yet the mechanisms behind the effect remain largely unclear and likely differ between diseases (119). The endotoxin tolerance induced by the TLR4 agonist LPS remains the prototypical model of innate immune tolerance (98, 120). While a single exposure to LPS induces a pro-inflammatory response, repeated or persistent exposure to high doses of LPS epigenetically induces a state of tolerance resulting in reduced pro-inflammatory cytokine production (121, 122).

While specific pathways and markers differ between the various programs in innate immune memory, they all display the same basic mechanisms (epigenetic, transcriptional, and metabolic). Mechanisms of epigenetic programming involve miRNAs, chromatin structure, DNA methylation (123, 124) and histone modification (125) and are central to this process (126, 127). There is a strong association between epigenetic changes and changes in gene expression patterns (123, 125).

There are reports on the association between DNA hyper- or hypomethylation and signaling pathways. Thus Kong et al. observed that multiple genes of the IL-12/IFN-γ signaling pathway (*IL12B*, *IL12RB2*, *TYK2*, *IFNGR1*, *JAK1*, and *JAK2*) were hypermethylated in patients with active TB, with decreased IFN-γ–induced gene expression and decreased IL-12–inducible upregulation of IFN-γ (124). Pacis et al. found a strong association between hypomethylated regions and transcription factors (TFs) from the NF-κB/Rel or the interferon regulatory factors (IRF) families (123). In Mtb-infected human DCs, TF binding motifs associated with NF-κB/Rel, AP-1, and IRF families were all significantly enriched within hypomethylated regions. Moorlag et al. found an association between trained immunity responses and enrichment for genes involved in the PI3K-Akt signaling pathway (125).

However, the molecular pathways linking chromatin remodeling to cellular signaling networks switching a transient signaling event into a long-lasting change and whether epigenetic changes are the cause of the modulation of signaling pathways or the other way around remain to a great extent to be explored. Pacis et al. observed that changes in gene expression in Mtb infected human DCs tend to occur prior to detectable changes in DNA methylation, supporting a model in which TF binding to enhancers leads to gene up-regulation followed by active demethylation, rather than vice versa (123).

### 3.3 Primary stimulants may induce tolerance or trained immunity *in vitro* depending on the experimental protocol

*In vitro*, innate immune memory can be established as either trained immunity with a heightened response to stimulation or tolerance with reduced ability to respond. This fate decision depends on factors such as activation state of the cells at the primary stimulation (113), antigen structure and conformation of the primary stimulus, delivery method, and receptor specificity of the stimulating microbial product.

Since experimental differences in the conditions for the stimulation and testing leads to great variation on the level of immune memory, several protocols have been established.

For β-glucan, BCG, and oxLDL, Bekkering et al. determined that the optimal experimental conditions for induction of trained immunity in macrophages are a training interval of 24 h followed by a resting time of 6 days (117, 128). Primary stimulation for 2 h, 4 h, or 24 h induced training but it was stronger after 24 h of training time. The trained phenotype then developed only after at least 3 days of resting, with a maximum occurring after 6 days (128). Interestingly, stimulation with β-glucan and BCG instead induced tolerance when cells were only briefly (24 h) left to rest before re-challenge (128). Likewise in a study by Yoshida et al., the expression of TNF was decreased 3 days after primary stimulation with LPS, while TNF expression was increased after 3 weeks of resting upon a secondary stimulation with LPS (129). An illustration of the importance of time for inducing innate tolerance is a study by Chavez-Galan et al. (69), where it took a minimum of 74 h of stimulation with LAM until LPS-induced TNF production was downmodulated.

In addition to stimulation and resting time, the concentration of the primary stimulation plays a role in inducing trained immunity or tolerance, with higher concentrations inducing tolerance while relatively lower concentrations leading to trained immunity. Thus, stimulation of human monocytes with a dose of 100 μg/ml of PAM3CSK4, a TLR2 agonist, tolerized the cells to respond with less IL-6 and TNF production upon restimulation with PAM3CSK4, while primary stimulation at lower doses, in the range of 1 μg/ml and below, induced a pattern of trained immunity with increased cytokine production upon restimulation (130). Even LPS, the prototype inducer of tolerance, may induce trained immunity when given at low doses (130–132). Thus, a high dose (100 ng/ml) of LPS induced tolerance upon restimulation with LPS, while a lower dose (10 ng/ml and below) induced trained immunity (130).

## 4 T cell hyporesponsiveness and Mtb glycolipids

T cell hyporesponsiveness, defined as decreased production of pro-inflammatory cytokines and/or increased production of IL-10, has been associated to Mtb glycolipids in a quite diverse set of situations. On the one hand this has been reported as a direct effect of Mtb glycolipids on T cells leading to a hyporesponse to other concurrent stimuli (133–135). On the other hand, this hyporesponse has been associated with a previous repeated and/or sustained immune stimulation in chronic infection or using *in vitro* models. The T cell hyporesponsiveness associated with prolonged/repeated stimulation may be caused by excessive stimulation of T cells with Mtb antigens or indirectly by tolerized APCs influencing adaptive immune responses (99, 100, 136–139), (**Figure 1** and **Table 3**).

**Table 3:** Induction of PBMC and T cell hyporesponsiveness by Mtb glycolipids.

Type of cells	Primary stimulation or simultaneous primary/secondary stimulation		Secondary stimulation		Effect	Modulatory effect	Ref
	Stimulant	Incubation time	Stimulant	Incubation time			
PBMC	LAM (3 and 30 μg/ml) or AM or D-arabino-D-galactan + PPD (1 μg/ml)	3 and 6 days	NA	NA	Proliferation → Proliferation ↓ Proliferation ↓↓ compared to no glycolipid	Downmodulation of PPD effect was induced by LAM and AM	(140)
T cell clones	LAM (3 and 30 μg/ml) + Influenza virus A	3 days	NA	NA	T cell proliferation ↓ compared to no LAM	Downmodulation of T cell proliferation was induced by LAM	(140)
Mouse CD4^+^ T cells	LAM (1 μM)	24 h	fixed BMMs pulsed with Ag85B (1 μg/ml)	48 h	T cell proliferation ↓ IL-2 ↓ compared to no LAM	Downmodulation of T cell proliferation was induced by LAM	(135)
Human PBMC	DAT	2 h	MTA (1 μg/ml)	5 days	Cell proliferation ↓ IL-2 ↓ IL-12 ↓ TNF ↓ IL-10 ↓ compared to no DAT	Downmodulation of cell proliferation and cytokine production in PBMC was induced by DAT	(141)
Human CD4^+^ or CD8^+^ T cells	DAT	2 h	MTA (1 μg/ml)	6 days	T cell proliferation ↓ CD25 ↓ CD69 ↓ compared to no DAT	Downmodulation of MTA effect was induced by DAT	(141)
Mouse spleen T cells	DAT (100 μM) + Mtb proteins (5.0 μg/ml)	24 h	NA	NA	T cell proliferation ↓ IFN-γ ↓ compared to no DAT	Downmodulation of Mtb protein effect was induced by DAT	(139)
Mouse spleen T cells	DAT (50 or 100 μM)	2 h	ConA (1.0 μ g/ml)	24 h	IL-2 ↓ IL-4 ↓ IFN-γ ↓ compared to no DAT	Downmodulation of ConA effect was induced by DAT	(139)

AM, Arabinomannan; ConA, Concanavaline A; DAT, Di-O-acyl- trehalose; LAM, Lipoarabinomannan; MTA, Mtb culture filtrate; PBMC, Peripheral blood mononuclear cells; NA, Not aplicable.

Symbols used: ↑ Increased production; ↓ Decreased production; → No difference; in relation to no Mtb glycolipid.

### 4.1 T cell hyporesponsiveness induced by concomitant T cell exposure to glycolipids

Direct exposure of CD4^+^ T cells, polyclonal and antigen-specific, to LAM during *in vitro* stimulation, has been shown to occur through insertion of LAM in the T cell membrane *via* lipid rafts (134, 142, 143). This insertion abrogates T cell receptor (TCR) signaling by blocking Zeta-chain-associated protein kinase 70 (ZAP-70) phosphorylation (134) and inhibiting the phosphorylation of both lymphocyte-specific protein tyrosine kinase (Lck), an Src family kinase, upstream of ZAP-70 and of the adaptor molecule linker of activation of T cells (LAT), downstream of ZAP-70, indicating that LAM blocks the proximal TCR signaling pathway (133, 134). Mahon et al. suggested that the insertion of LAM in the T cell membrane allows other components of the LAM molecule, such as the mannose cap, to interfere with the TCR complex perturbing its function (134). Athman et al. proposed an additional mechanism based on *in vitro* and *in vivo* studies, where LAM is transferred to T cells through membrane vesicles released from infected macrophages, which induces GRAIL, leading to inhibition of CD4^+^ T cell activation with reduced production of IL-2 and of cell proliferation (144).

### 4.2 T cell hyporesponsiveness indirectly induced through alteration of the APCs

The stimulation/activation of Mtb-specific T cells during the immune response to Mtb depends on the presentation of Mtb-antigens to T cells *via* classical MHC and CD1 molecules (145, 146). Using a mouse model of Mtb infection, Reiley et al. showed data suggesting that during late stages of the chronic infection APCs lose the ability to stimulate naive T cells (147). Studies *in vitro* also revealed that exposure of DCs to BCG triggers DCs to enhance IL-10 and diminish IL-12 production, inducing naive T cells to develop into IL-10-producing T cells in a dose-dependent manner (148). This suggests that BCG vaccination might result in the development of IL-10-producing DCs and IL-10-producing T cells that could contribute to restricting overt inflammation (148).

One mechanism responsible for the delayed/reduced T cell response through alteration on the APCs is by antigenic evasion. Several pathways are involved in the inhibition/reduction of Mtb-antigens presentation by APCs to T cells. These strategies differentially affect peptide and lipid antigens as shown by Hava et al. (149). Mtb infection causes rapid DC maturation and consequently delays presentation of Mtb-antigenic peptides to T cells *via* MHC II molecules. Interestingly, this antigenic evasion was not detected for lipid antigens (149). Other strategies are used by Mtb to delay/reduce the presentation of peptide antigens by MHC II and consequently delay/reduce CD4^+^ T cell activation; namely through the inhibition of the endosomal sorting complex required for transport (ESCRT) machinery by Mtb EsxH (150). In addition, Mtb glycolipids, in particular Mtb 19-kDa lipoprotein, were shown to inhibit MHC II expression and antigen processing in murine macrophages, with a subsequent decreased presentation of Mtb-antigenic peptides to T cells (88, 151, 152). Noss et al. showed that this occurs *via* binding of 19-kDa to TLR2 (151, 152).

Presentation of mycobacterial glycolipids by CD1b molecules to T cells has been shown to occur for all glycolipids studied so far, namely LAM, PIM and LM (110, 153–157), sulfoglycolipids (110, 112, 154), GroMM (110) (111), GMM (158, 159), and mycolic acids (110, 160–163). GMM has also been shown to be presented by CD1c in humans and nonhuman primates (158).

Upon activation through the TCR, CD1-restricted T cells were shown to secrete pro-inflammatory cytokines such as IFN-γ (112, 164), and to kill Mtb-infected APCs (112, 154, 165). This contributes to reduced mycobacterial proliferation and survival. In several experiments, the role of CD1b was confirmed by adding anti-CD1b blocking antibodies, which resulted in substantial inhibition of the T cell response (109, 110, 164). CD1-restricted T cells recognizing mycolic acids from patients with active TB were shown to be expanded in TB patients at diagnosis but were not detected in uninfected BCG-vaccinated controls, similar to conventional MHC-restricted T cells (160). GroMM was found to be presented by Mtb-infected DCs, demonstrating that the antigen is available for presentation during natural infection. In contrast, a tetramer study with Peruvian subjects found no significant differences in the numbers of T cells recognizing CD1b tetramers loaded with mycolic acid or GMM between subjects with Mtb exposure, latent Mtb infection or active TB (166). This potentially indicates that some Mtb glycolipids are more likely than others to induce T cell responses during infection.

Similar to the hyporesponsiveness to peptide antigens from Mtb that are presented to TCRs in the context of MHC molecules, one mechanism responsible for the induction of hyporesponsiveness of CD1 restricted T cells may be through alteration of the APCs and their presentation of Mtb glycolipids by CD1b molecules to T cells. Virulent Mtb strains are able to suppress the action of CD1 restricted T cells, by downmodulating CD1 expression on APCs (167). The Mtb glycolipid DAT, that is recognized by CD1b (168), was shown to reduce antigen-induced proliferation of CD4^+^ and CD8^+^ T-cell subsets *in vitro* using PBMCs. The effect was associated with decreased expression of the T-cell surface activation markers CD25 and CD69, and reduced production of IL-2, IL-12, TNF and IL-10 (141). This effect was also observed, by the same group, for mouse T cells (138). Studies *in vitro* showed that DAT has an inhibitory effect on proliferation and mRNA expression of IL-2 and IFN-γ in antigen-stimulated T cells from Mtb-infected mice. This effect involved down-modulation of the di-acyl glycerol-dependent activation of the MAPK-ERK1/2 pathway, one of the crucial signaling pathways leading to adaptive cellular immune responses against Mtb infection (139).

Most studies analyzing the T cell response to glycolipids using blood samples compared the response of cells from individuals with active TB and healthy controls. A few studies added to this data on the immune response of individuals that are positive for PPD and/or IGRA but without signs of active diseases. Ulrich et al. showed that when nonadherent PBMCs were stimulated with Mtb total-lipid extract, the proliferative response of PPD-positive individuals was enhanced compared to the ones with active TB as well as healthy controls (**Table 3**). In addition, the same authors presented data supporting the involvement of CD1-restricted CD4^+^ T cells on this proliferative response and IFN-γ production (109). Likewise, *ex vivo* T cell responses to the Mtb lipid antigen GroMM were detected in the blood of latently infected individuals, as well as BCG vaccinated, but not in patients with active TB potentially indicating a functional rather than numeral enrichment (111). Recent data from our group showed that T cells from individuals with latent TB or active TB present a distinct response profile to LAM and PIM (18). The overall production of cytokines in response to PIM was clearly reduced in individuals with active TB compared to healthy controls, strengthening the notion of a hyporesponse during the course of active disease. In contrast, T cells from PPD/IGRA+ individuals responded to PIM with increased production of several pro-inflammatory cytokines, namely IL-6 and TNF (18).

### 4.3 T cell exhaustion

Although the mechanisms for downmodulation of restricted T cells by Mtb glycolipids in individuals with Mtb infection have not been explored in detail, available data suggest that they have similarities to the T cell exhaustion caused by stimulation with Mtb peptides/proteins (169). T cell exhaustion is a phenomenon well known in human TB/Mtb infection (136, 137, 170, 171) and experimental Mtb or BCG infection in mice (172–174).

Human Mtb*-*specific CD8^+^ T cells display decreased proliferation and production of IL-2, IFN-γ, and TNF in response to Mtb antigens such as CFP-10 and ESAT-6 in active TB compared to latent TB (170). In mice, infection with Mtb (172) or BCG (175) resulted in a gradual loss of TNF and IL-2 production by individual T cells upon persistent antigen exposure (172, 175), as well as a decreased proliferation of IFN-γ producing T cells (175). T cell exhaustion is more pronounced in patients with active than with latent TB. In patients with active TB, a hyporesponsive T cell phenotype with decreased immune proliferation and decreased cytokine production induced by Mtb antigens and mitogens was associated with DNA hypermethylation of several immune genes and pathways, including the IL-2/STAT5, TNF/NF-κB, and IFN-γ signaling pathways (137).

Negative regulatory pathways such as immunoregulatory cytokines have been shown to be involved in the exhaustion of T cells (176). Exhausted T cells have been described to express immune checkpoint inhibitory receptors including programmed death 1 (PD-1), the T cell immunoglobulin and mucin domain–containing-3 (Tim-3) receptor, lymphocyte-activation gene 3 (Lag-3), and cytotoxic T-lymphocyte-associated protein 4 (CTLA-4), which interact with various ligands to activate negative regulatory pathways (172, 176, 177). In Mtb-infected mice, exhausted T cells (CD4^+^ as well as CD8^+^) express PD1, Tim-3, and Lag-3, and show low production of IL-2, IFN-γ, and TNF but increased production of IL-10 (172). In humans, PD-1^+^ T cells are increased in patients with active TB (178, 179) and T cell stimulation with Mtb antigens increased PD-1^+^ T lymphocytes in peripheral blood and pleural fluid from patients with active TB (180). In individuals with active TB, Jean Bosco et al. observed that stimulation *ex vivo* of CD4^+^ CXCR5^+^ T cells with Mtb antigen, induced expression of Tim-3 and PD-1 (181). However, it is noteworthy that Sande et al. found that although LAM-treated CD4^+^ T cells exhibited the expected decrease in proliferation, there was no significant increase in the expression of the exhaustion markers PD-1, CTLA-4, Lag-3, or Tim-3 compared with nontreated T cells (135).

Reverting exhaustion by blocking inhibitory receptors such as the main exhaustion markers PD-1 or Tim-3 would be tempting to explore as a means to improve the protection against disease (8). Indeed, reversal of T cell exhaustion can be obtained in chronically Mtb infected mice by IL-2 treatment (175) or Tim-3 blockade with anti-Tim-3 monoclonal antibody, resulting in reduced bacterial load (172). Also, in individuals with active TB, Jean Bosco et al. observed that blockade of Tim-3 and PD-1 restored the proliferation and cytokine secretion potential of exhausted T cells (181).

However, Kauffman et al. recently described that Mtb infected rhesus macaques treated with anti-PD-1 monoclonal antibody developed a more severe disease and higher granuloma bacterial load compared with isotype control-treated monkeys (182). In granulomas of animals treated with anti-PD-1, pro-inflammatory cytokines were increased, which also correlated with elevated bacterial load. Therefore, it appears that negative immune regulation of T cells is needed to control Mtb infection by dampening detrimental immunopathology to counteract the progression of the disease.

## 5 Discussion

There is increasing evidence that mycobacterial glycolipids play a major role in the dampening of the immune response in Mtb infection involving several mechanisms, especially in myeloid cells such as macrophages and DCs, but also in T cells. Depending on cell type, the downmodulation may be manifested as impaired immune cell activation and differentiation but may also show patterns of innate immune tolerance. In myeloid cells, the downmodulation induced by LAM/PIM on monocytes and macrophages mainly results in reduced secretion of pro-inflammatory cytokines and of the granulomatous inflammatory response, while the effect on DCs also contributes to the tolerization of T cells.

In *in vitro* experiments several factors determine the outcome of the encounter between Mtb glycolipids and host cell receptors. For example, triggering murine or human TLR2 leads to divergent responses (183). Additionally, some C-type lectins pathways, such as those of Mincle and Dectin-2 are strongly active in murine BMDCs, but poorly active in human monocyte-derived DCs (36). The cell type is also important; for instance, murine BMDCs are strongly biased toward C-type lectin signaling whereas murine BMDMs are biased towards TLRs, although both types of receptors are expressed on these cells (36). The preparation and purity of the cells and glycolipids may also affect the experimental outcome (77, 184).

There should also be caution in extrapolating data from *in vitro* experiments to the outcome of the early encounter of the whole bacterial organism *in vivo*, where we do not know the importance of the combination of stimuli and the receptor equipment of the cell(s) that are first encountered in the body by the minute number of bacteria arriving in the lung. In addition to the fact that Mtb exposes surface lipoglycans such as LAM and LM at its cell envelope (185), Mtb glycolipids are also released from the envelope of Mtb and are detected in the endosomal compartments of infected cells or in extracellular vesicles (144, 186, 187). Thus, the immunomodulatory properties of LAM are not restricted to intact Mtb, but also apply to circulating LAM released from infected APCs in exosomes or apoptotic vesicles (188, 189). This might actually be the main mechanism by which LAM exerts its immunomodulatory properties on infected or bystander cells. For example, the immunosuppressive effect by isolated LAM observed *in vitro* could have its counterpart *in vivo*, with extracellular LAM exerting the effect, while LAM on intact bacteria may be presented in a way that does not allow the induction of this hyporesponsiveness.

The dampened inflammatory response of myeloid cells in individuals infected with Mtb could be caused by a tolerant state of the cells, where the Mtb infection serves as the primary stimulant, possibly by the presentation of Mtb glycolipids, resulting in the downmodulation by a secondary stimulant such as LAM or PIM seen in *ex vivo* experiments (18, 108). The possibility of innate immune tolerance being involved in the containment of Mtb during latency, where the immune system is continuously exposed to Mtb antigens including glycolipids, remains to be explored.

It has become increasingly clear that an immune response balancing between activation and tolerance is key to controlling Mtb infection (182). Both the adaptive and the innate immune response to fight invading pathogens may result in inflammation and tissue damage, with an overproduction of pro-inflammatory cytokines. Innate immune tolerance results in hyporesponsiveness to microbial components that induce inflammation by various mechanisms (190). A balance between the effects of inflammation and tolerance may thus result in a steady-state, where the pathogen survives but remains under control without damage to the host. Identifying the mechanisms responsible for the containment of the Mtb infection in latency, in whom the infection is controlled (191), may be a means to elucidate factors of importance in this balance.

Recent data (18) indicate that downmodulation of pro-inflammatory responses in myeloid cells may be stronger in latent TB compared with active TB. In latent TB, the immune profile is thought to represent a more protective pattern than in active TB (191). A less pro-inflammatory myeloid immune response has been associated with increased resistance to TB in pre-adolescent children (192). The downmodulation may play a role in the protection from excessive production of pro-inflammatory cytokines and tissue damage as part of the control of the infection (119). Thus, the balance between activation and inhibition of pro- and anti-inflammatory cytokines by glycolipids may dictate the host response to Mtb infection (67), from equilibrium in latent infection to the lung injury in pulmonary TB to disseminated disease.

Further studies of tolerance to Mtb glycolipids may provide relevant information to identify, among the individuals with latent TB, those at higher risk to develop active disease. A method that identifies individuals with latent TB that are at high risk of developing active TB is urgently needed, for proper treatment, and also to avoid unnecessary treatment of those at low risk. It would be of particular value for immune-compromised persons, including the increasing group of patients receiving immune-modulating treatment. More hypothetically, further studies of tolerance induced by Mtb glycolipids may unravel mechanisms for the rational design of personalized immunotherapy, as an add-on to existing antimicrobial therapies, by dampening detrimental immunopathology and counteracting progression of the disease.

## Author contributions

GK, MC-N and CS decided on the general content of the manuscript, collected the data, and summarized and structured the information. GK, MC-N, CS and JN wrote the different chapters. ZM provided important contributions on the signalling pathways, text and figures. All authors contributed to the article and approved the submitted version.

## Funding

This work was supported by grants from the Swedish Research Council (2019-04663 and 2020-03602) and the Swedish Heart- and Lung Foundation (20180386 and 20200194) to GK and Swedish Research Council (2021–03706) and the Swedish Medical Association (SLS-934363) to CS. From the Fondation pour la Recherche Médicale (Equipes FRM DEQ20180339208 to JN), MSDAVENIR (grant Fight-TB to JN), the French National Research Agency (ANR-19-CE15-0012-01 to JN) and the European Union’s Horizon 2020 research and innovation program under grant agreement No 847762 (to JN).

## Conflict of interest

The authors declare that the research was conducted in the absence of any commercial or financial relationships that could be construed as a potential conflict of interest.

## Publisher’s note

All claims expressed in this article are solely those of the authors and do not necessarily represent those of their affiliated organizations, or those of the publisher, the editors and the reviewers. Any product that may be evaluated in this article, or claim that may be made by its manufacturer, is not guaranteed or endorsed by the publisher.
